# Lyme Borreliosis Incidence Across Europe, 2015–2023: A Surveillance-Based Review and Analysis

**DOI:** 10.1177/15303667251363125

**Published:** 2025-10-03

**Authors:** Alexander Davidson, Julie Davis, Gordon Brestrich, Jennifer C. Moisi, Luis Jodar, James H. Stark

**Affiliations:** ^1^ Global Vaccines Medical Affairs, Pfizer Research & Development, New York, New York, USA.; ^2^ Global Vaccines Medical Affairs, Pfizer Research & Development, Cambridge, Massachusetts, USA.; ^3^ Global Vaccines Medical Affairs, Pfizer Research & Development, Berlin, Germany.; ^4^ Global Vaccines Medical Affairs, Pfizer Research & Development, Paris, France.; ^5^ Global Vaccines Medical Affairs, Pfizer Research & Development, Collegeville, Pennsylvania, USA.

**Keywords:** Lyme borreliosis, incidence, surveillance, Europe, tick-borne disease

## Abstract

**Background:**

Lyme borreliosis (LB) is the most common tick-borne disease in Europe. Incidence is increasing partly due to climate change and changing human behaviors. This review compiled updated national European LB surveillance data to assess recent incidence trends from 2015 to 2023.

**Methods:**

Publicly available LB surveillance data sources were identified for 29 European countries. Cases were collected from reports and dashboards. Estimates of population-based incidence were calculated. Countries were categorized based on their case classification criteria and geographic region. Subnational regions were classified as high-incidence areas if they reported ≥10 cases per 100,000 population per year (PPY) over a three-year period. The percentage of people residing in these high-incidence regions was calculated.

**Results:**

An average of 132,000 LB cases were reported to surveillance systems annually. Countries that included both erythema migrans and laboratory-confirmed LB cases in their reporting had the highest incidence rates—particularly Estonia, Finland, and Slovenia, exceeding 100 cases per 100,000 PPY. Overall, variation of incidence at the subnational level was observed, showing incidence at the national level can mask subnational differences in rates. Temporary decreases in incidence were observed during 2019–2021, but incidence increased from 2021 to 2023. A minimum of 223 million people, equating to 30% of the entire European population and 51% of those living in a European country with available subnational data, live in an area with high LB incidence.

**Conclusion:**

High LB incidence across Europe was observed from 2015 to 2023, particularly in parts of Northern, Eastern, and Western Europe. For nearly 70% of countries with recently published LB surveillance, incidence increased an average of 36% in the last two years of reporting. These findings emphasize the need for continued monitoring of LB across Europe and new public health prevention tools, such as enhanced tick prevention campaigns, improved education among health care providers, and effective vaccines.

## Introduction

Lyme borreliosis (LB) is a tick-borne disease (TBD) caused by infection with the bacterium *Borrelia burgdorferi* sensu lato (*Bb*) through the bite of an infected tick (Stanek and Strle, 2018). In Europe, LB is the most common vector-borne disease, and a past review of LB surveillance data reported an average of nearly 129,000 to national surveillance systems annually (Burn et al., 2023).

Clinical presentation of LB varies greatly, and the disease can present as different clinical manifestations. Early localized disease is most common, affecting 80–94% of all cases, and typically presents as erythema migrans (EM), a localized bullseye-like rash that appears days or weeks after a tick bite (Stanek and Strle, 2018; Geebelen et al., 2019; Hofhuis et al., 2015). Generalized flu-like symptoms, such as fever, arthralgia, and fatigue, may present here as well (Wormser, 2006; Stanek and Strle, 2018). Without the appearance or identification of EM or without proper antibiotic treatment, early disseminated disease may develop, resulting in conditions such as Lyme neuroborreliosis (LNB) or Lyme carditis. LB can further manifest in late disseminated disease, with conditions such as Lyme arthritis or acrodermatitis chronica atrophicans (Steere et al., 2016; Bobe et al., 2021). While early localized disease is often diagnosed clinically due to the unique EM presentation, disseminated disease is rarer and confirmed by laboratory testing (Steere, 2001).

Differences in disease presentations and diagnostic tests can make it difficult to evaluate trends in LB incidence, particularly between countries. First, there is significant heterogeneity in the diagnostic tests utilized (*e.g.,* direct vs. indirect testing or tests with varying accuracy) among European countries to detect LB cases (European Centre for Disease Prevention and Control, 2016). Second, while uniform LB case definitions have been proposed using a combination of clinical and laboratory diagnostic criteria, such as by The European Concerted Action on Lyme Borreliosis (EUCALB), these have not been widely adopted (Stanek et al., 2011). Reviews of European-wide LB surveillance systems show that where routine surveillance is conducted, case definitions for what is notifiable vary, from specific manifestations only to clinical and/or laboratory diagnostic requirements (Blanchard et al., 2022; Nagarajan et al., 2023). This not only limits comparisons of LB across countries but also underestimates the number of LB cases in each country (Burn et al., 2023; van den Wijngaard et al., 2017a).

In 2018, the European Center for Disease Prevention and Control (ECDC) began collecting case data on LNB using a standard case definition (European Union, 2018). Though LNB is the most common disseminated manifestation of LB, it is still rare with several studies estimating it to affect about 3% of cases in European population samples (Brestrich et al., 2024b; Geebelen et al., 2019; Hofhuis et al., 2015). Across the European Union (EU) and European Economic Area (EEA), 592–1497 LNB cases have been reported annually between 2018 and 2023 (European Centre for Disease Prevention and Control, 2024). While ECDC’s standardized aggregated LB surveillance allows comparability of LNB, it does not include non-EU or EEA countries that conduct surveillance nor include other manifestations, therefore significantly underestimating the burden of LB.

Despite its limitations, LB surveillance is beneficial to assess temporal trends in disease. The most recent review of LB surveillance data from 25 European countries from 2005 to 2020 summarizes historical trends and found increasing incidence over time (Burn et al., 2023). It has been postulated that incidence will continue to increase given the expanding ranges of ticks and host reservoirs due to climate change and changing human behaviors that put people in contact with ticks more often (Janova, 2019; Semenza and Suk, 2018). Therefore, it is essential to continue to monitor and aggregate LB surveillance data to identify areas where the disease is spreading and incidence continues to increase. This updated review of LB surveillance data across Europe builds upon what was previously found by including data for additional countries and the most up-to-date years (Burn et al., 2023). It also emphasizes the heterogenous LB case definitions that national surveillance systems report out and highlights a way to make these data more comparable. These insights can help inform future prevention and intervention strategies in at-risk populations in Europe.

## Methods

Publicly available national LB surveillance data were searched for and identified in 29 of the 47 countries in the Europe Region of the United Nations (https://unstats.un.org/unsd/methodology/m49/) (43 plus the U.K. countries of England, Northern Ireland, Scotland, and Wales). Where case data from government public health agency websites and dashboards were identified, data were downloaded and reviewed for all available years. Sources for country-level case data and case information can be found in Supplementary Table S1. Non-English surveillance reports were translated using *DeepL Translator Pro 2021*, Microsoft Word, or Google Translate.

### Reporting case classifications

Surveillance agencies across Europe have varying criteria for LB case notification, which may or may not match case definitions used in their surveillance reports (Supplementary Table S1). Therefore, five reporting case classification categories were created to describe the types of LB cases published in identified reports and dashboards and to bolster comparability of national incidence across countries. The five categories ordered from the most to least inclusive, meaning capturing the broadest LB clinical presentations to the most restrictive, are: “EM or laboratory-confirmed LB”—countries whose surveillance-reported cases can be clinically diagnosed EM or any other laboratory-confirmed LB case; “EM only”—countries whose surveillance-reported cases are only clinically diagnosed EM; “Laboratory-confirmed LB”—countries whose surveillance-reported cases are any laboratory-confirmed LB case; “Laboratory-confirmed, disseminated LB”—countries whose surveillance-reported cases are any laboratory-confirmed LB case with clinical evidence of a disseminated manifestation; and “LNB only”—countries whose surveillance-reported cases are laboratory-confirmed LNB cases only.

### LB incidence synthesis

Case data from the 29 countries identified from 2015 to 2023 were extracted into Microsoft Excel. To derive incidence estimates, annual population statistics for each country for each year of available data from national statistics websites were retrieved (Supplementary Table S1). Eurostat was used when nationally provided estimates were unavailable or insufficient. Population-based incidence estimates were calculated as the number of cases per 100,000 population per year (PPY) for each country and subnational region for which data were available.

National LB incidence trends by country were described, grouping countries by reporting case classifications and European region. For the purpose of this analysis, Europe was categorized into four main regions: Northern Europe (subdivided into the United Kingdom and Ireland, the Nordic region, and the Baltic region), Western Europe, Eastern Europe (subdivided into East Central and Eastern), and Southern Europe (World Health Organization, 2024).

Subnational regional data were extracted where available. Data were extracted at the region types and levels provided, which included the first, second, and third levels of the Nomenclature of territorial units for statistics (https://ec.europa.eu/eurostat/web/nuts/) or International Organization for Standardization 3166–2 country subdivisions (https://www.iso.org/).

LB incidence estimates were calculated over the most recent three years for which case and population data were available for each country and subnational region.

### Percent population living in regions with high LB incidence

The percentage of European countries’ populations that live in subnational regions with high LB incidence was also calculated. Since there is no accepted European-wide threshold for high LB incidence, the United States Centers for Disease Control and Prevention definition was used, which is an area reporting ≥10 cases per 100,000 PPY over any three consecutive year period (Centers for Disease Control and Prevention, 2021). For this part of the analysis, 19 countries with subnational surveillance data and a more robust reporting case classification than “LNB only” were included. For each included country, the populations from high-incidence subnational regions were summed (“numerator”), divided by the sum of all subnational regions with surveillance data (“denominator”), and multiplied by 100. The resulting percentage represents the country’s population living in an area of high LB incidence.

Furthermore, two estimations for the percentage of the European population living in areas with high incidence of LB were calculated. First, to estimate the percentage of the population living in a high-incidence region of Europe with publicly available LB surveillance data, the sum of “numerators” from each country’s percent was divided by the sum of the “denominators” and multiplied by 100. Second, to derive an estimate of the minimum number of people across Europe living in regions with high incidence of LB, the sum of “numerators” from each country’s percent was divided by the sum of the 2023 population estimates for the 47 European countries in the UN Europe region and multiplied by 100.

All analyses were performed in Microsoft Excel. Maps were generated using QGIS, version 3.32.1.

## Results

National LB surveillance case data were identified and extracted for 29 countries (Table 1). Case data were identified for 11/11 (100%) countries in Eastern Europe, 11/13 (85%) in Northern Europe, 4/9 (44%) in Western Europe, and 3/14 (21%) in Southern Europe (Table 1). From 2015 to 2023, an average of 131,966 (range: 98,252–172,805) LB cases were reported annually from countries with surveillance systems that made data publicly available (Supplementary Table S2).

**Table 1.** Results of Search for Publicly Available National Lyme Borreliosis Surveillance in 47 European Countries and Case Classification Categorization of Reported Cases, by Country

Region	LB surveillance identified and extracted	Country	Case classification of reported cases
Eastern Europe			
*** ***Eastern	Yes	Belarus	EM or lab-confirmed LB
		Bulgaria	Lab-confirmed LB
		Romania	EM or lab-confirmed LB
		Russia	EM or lab-confirmed LB
		Moldova	Lab-confirmed LB
		Ukraine	EM or lab-confirmed LB
East Central	Yes	Czechia	EM or lab-confirmed LB
		Hungary	EM or lab-confirmed LB
		Poland	EM or lab-confirmed LB
		Slovakia	EM or lab-confirmed LB
		Slovenia	EM or lab-confirmed LB
Northern Europe			
Baltic region	Yes	Estonia	EM or lab-confirmed LB
		Latvia	Lab-confirmed LB
		Lithuania	EM or lab-confirmed LB
Nordic region	Yes	Denmark	LNB only
		Finland	EM or lab-confirmed LB
		Norway	Lab-confirmed, disseminated LB
			
	No	Iceland	—
		Sweden	—
United Kingdom and Ireland	Yes	England	Lab-confirmed LB
		Ireland	LNB only
		Northern Ireland	Lab-confirmed LB
		Scotland	Lab-confirmed LB
		Wales	Lab-confirmed LB
Western Europe			
	Yes	Belgium	EM only^a^
		France	EM or lab-confirmed LB
		Germany	EM or lab-confirmed LB
		Switzerland	EM only
	No	Austria	—
		Liechtenstein	—
		Luxembourg	—
		Monaco	—
		Netherlands	—
Southern Europe			
	Yes	Croatia	EM or lab-confirmed LB
		Portugal	EM or lab-confirmed LB^b^
		Serbia	EM or lab-confirmed LB
	No	Albania	—
		Andorra	—
		Bosnia and Herzegovina	—
		Greece	—
		Holy See	—
		Italy	—
		Malta	—
		Montenegro	—
		North Macedonia	—
		San Marino	—
		Spain	—

A total of 47 countries of the Europe Region of the United Nations (43 countries plus the United Kingdom’s England, Northern Ireland, Scotland, and Wales) were considered for the search. For analysis purposes, countries are categorized by WHO European regions. Where publicly available LB surveillance data were identified, reported cases were classified into five categories: (1) EM or laboratory-confirmed LB—countries whose surveillance-reported cases can be clinically diagnosed EM or any other laboratory-confirmed LB case; (2) EM only—countries whose surveillance-reported cases are only clinically diagnosed EM; (3) Laboratory-confirmed LB—countries whose surveillance-reported cases are any laboratory-confirmed LB case; (4) Laboratory-confirmed, disseminated LB—countries whose surveillance-reported cases are any laboratory-confirmed LB case with evidence of a disseminated manifestation; and (5) LNB only–countries whose surveillance-reported cases are LNB cases only.

^a^ Reports from Belgium’s sentinel surveillance system also include data on positive LB laboratory tests and possible LB-related hospitalizations. However, these data are not confirmed to be LB cases and therefore not included in this analysis.

^b^ Through 2018, Portugal reported cases that were EM or laboratory-confirmed LB. Since 2018, Portugal no longer publicly reports LB surveillance data, although it does still conduct surveillance on LNB cases only.

EM, erythema migrans; Lab, laboratory; LB, Lyme borreliosis; LNB, Lyme neuroborreliosis.

Reporting case classification categories differed across the 29 countries. Seventeen (59%) countries have the broadest case classification, including cases that are EM or laboratory-confirmed, 7 (24%) include laboratory-confirmed cases only, 2 (7%) include cases that are EM only, 1 (3%) includes cases that are laboratory-confirmed with disseminated manifestations only, and 2 (7%) have the most restrictive case classification, reporting laboratory-confirmed LNB cases only (Fig. 1, Table 1).

**FIG. 1.**
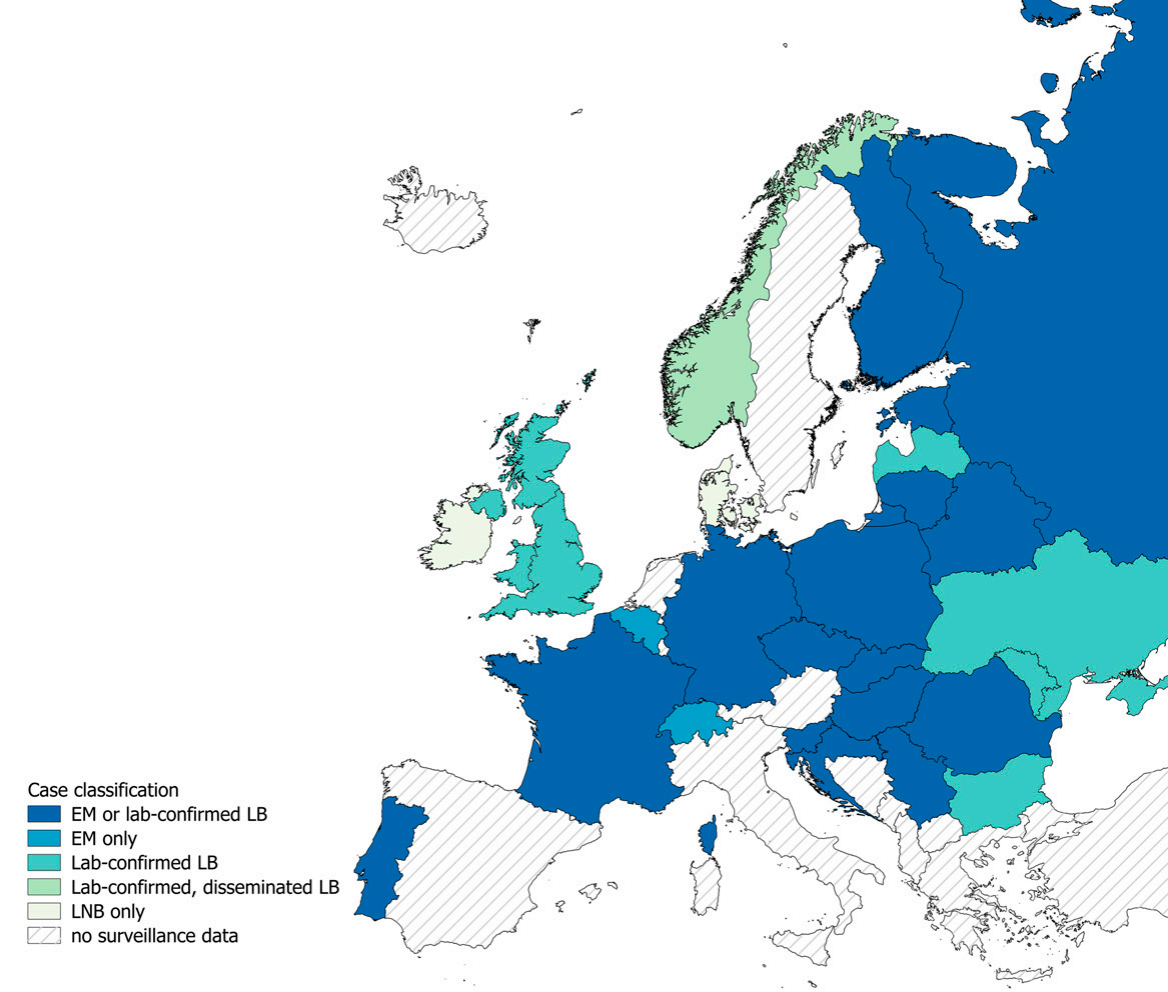
Case classification categorizations of reported Lyme borreliosis cases in national surveillance systems across Europe. Map represents the case classifications of LB cases reported national surveillance data for European countries identified from our search methodologies. Links to surveillance systems are found in Supplementary Table S1. The reported case classification system is as follows: *EM or laboratory-confirmed LB*—countries whose surveillance-reported cases can be clinically diagnosed EM or any other laboratory-confirmed LB case; *EM*—countries whose surveillance-reported cases are only clinically diagnosed EM; *laboratory-confirmed LB*—countries whose surveillance-reported cases are any laboratory-confirmed LB case; *laboratory-confirmed, disseminated LB only*—countries whose surveillance-reported cases are any laboratory-confirmed LB case with evidence of a disseminated manifestation; and *LNB*—countries whose surveillance-reported cases are laboratory-confirmed LNB cases only. EM, erythema migrans; Lab, laboratory; LB, Lyme borreliosis; LNB, Lyme neuroborreliosis.

Twenty-six countries have passive surveillance systems with reporting from clinicians and/or laboratories while three (Belgium, France, Switzerland) have physician-reported sentinel surveillance systems (Supplementary Table S1). Additionally, 26 countries continue to report annual LB surveillance data as of 2022 or 2023, while three countries no longer do; Northern Ireland and Serbia stopped publicly reporting data in 2017 and Portugal in 2018. Complete tabulated LB case and incidence data for the 29 countries reporting data from 2015 to 2023 are available in Supplementary Table S2.

### 2015 to 2023 LB incidence trends

Overall, the highest LB incidence estimates ≥100 per 100,000 PPY were observed in countries with broader reporting case classifications (EM & laboratory-confirmed LB and EM only) compared with narrower ones (Fig. 2). When focusing on the 17 countries with the broadest LB reporting case classification, countries in Northern, Western, and East Central Europe generally have higher LB incidence estimates from 2015 to 2023 compared with those in Eastern and Southern Europe (Fig. 2a, b). Eastern and Southern European countries report EM and laboratory-confirmed LB and incidence was <20 per 100,000 PPY for all years except for Belarus in 2022 and 2023 (Fig. 2b). In contrast, the nine countries of Northern, Western, and East Central Europe with the same case classification had incidence estimates ranging from 25 to 365 per 100,000 PPY except for Slovakia prior to 2022 (Fig. 2a).

**FIG. 2.**
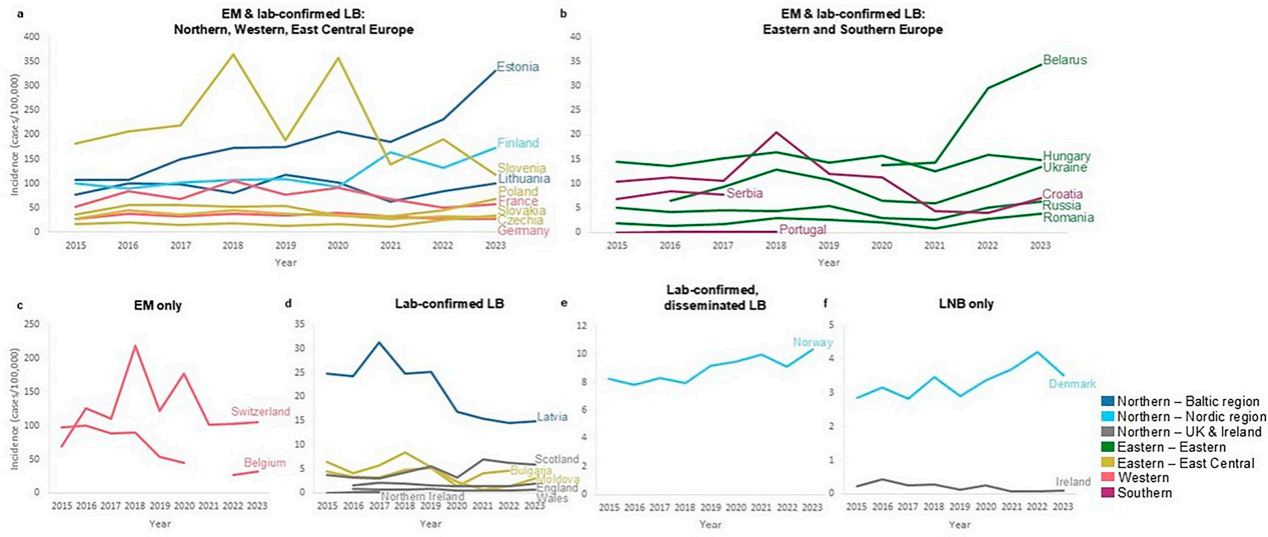
Incidence of surveillance-reported Lyme borreliosis for countries in Europe, by reporting case classification, 2015–2023. Incidence estimates displayed in number of LB cases per 100,000 PPY. Incidence estimates shown by determined reporting LB case classification: **(a—b)** EM and laboratory-confirmed LB, **(c)** EM only, **(d)** laboratory-confirmed LB, **(e)** laboratory-confirmed, disseminated LB, **(f)** LNB only. *Y*-axis scale for incidence estimates differ in **(a—f)**. Color of the trend line is determined by the country’s European region. Gaps in data occur when no data are reported for year(s). Northern Ireland and Serbia stopped reporting LB surveillance data in 2017 and Portugal in 2018. Belarus data available starting in 2020. Tabulated data are provided in Supplementary Table S2. EM, erythema migrans; Lab, laboratory; LB, Lyme borreliosis; LNB, Lyme neuroborreliosis; U.K., United Kingdom; PPY, population per year.

Among countries with more restrictive reporting case classifications, similar trends were identified. Switzerland and Belgium in Western Europe reported EM incidence estimates >20 per 100,000 PPY for all years, exceeding estimates in Eastern and Southern European countries with broader case classifications (Fig. 2b, c). Latvia, in the Baltic region, reported more than twice the laboratory-confirmed LB incidence than two Eastern European and the four U.K. countries each year (Fig. 2d). Norway, in the Nordic region, reported 7.8–10.3 disseminated cases per 100,000 PPY and Denmark 2.9–4.2 LNB cases per 100,000 PPY. These estimates include less inclusive reporting case classifications but still exceed incidence estimates from countries with more inclusive classifications across in Eastern and Southern Europe and across the U.K. and Ireland (Fig. 2e, f).

### LB incidence over the last 3 years

Over the three most recent years of data, similar trends in incidence of LB across Europe were observed. Countries with broader case classifications in Western, East Central, and the Nordic and Baltic regions of Northern Europe reported the greatest incidence estimates during 3-year period (Fig. 3). Of the 26 countries that continue to report annual surveillance data, 18 (69%) had a ≥10% (mean = 36%) increase in incidence over the latest two years reported, six had stable incidence (<10% increase or decrease in incidence), and two had a ≥10% decrease in incidence (Fig. 2, Supplementary Table S2).

**FIG. 3.**
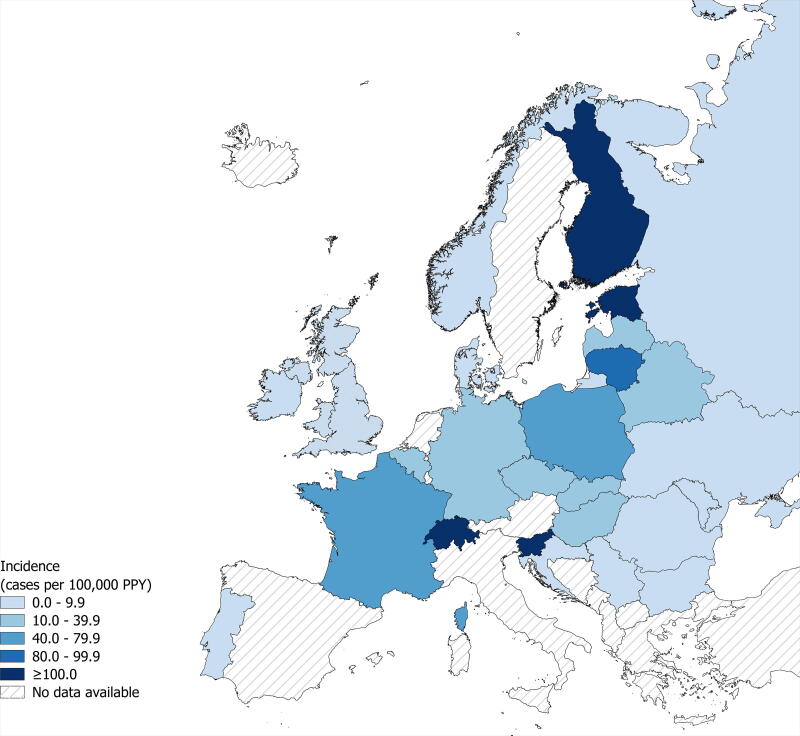
Incidence of surveillance-reported Lyme borreliosis in countries in Europe during the most recent three-year period. Incidence of LB (cases per 100,000 PPY) during the most recent three-year period from national surveillance systems. Presented national LB surveillance data is of the most recent three years of data available for European countries identified from search methodologies and includes all reporting case classifications as described in Table 1 and Fig. 1. Most recent three years of data vary by country. LB, Lyme borreliosis; PPY, population per year.

Subnational variations in incidence were observed within larger countries, regardless of reporting case classification (Fig. 4, Supplementary Figs. S1 and Figs. S2). In the Nordic countries, the southern regions of Finland and Norway reported higher incidence during the three-year period than their northern regions (Fig. 4). In Western Europe, the highest incidence (≥100 per 100,000 PPY) was seen in southern, southcentral, and eastern areas of France and the eastern states of Germany (Fig. 4). In Poland, the central regions and the southeastern Lower Silesia had the lowest incidence estimates ranging from 24.5 to 34.0 per 100,000 PPY, while the remaining regions, particularly in the west, had incidence ≥40 per 100,000 PPY (Fig. 4, Supplementary Table S2).

**FIG. 4.**
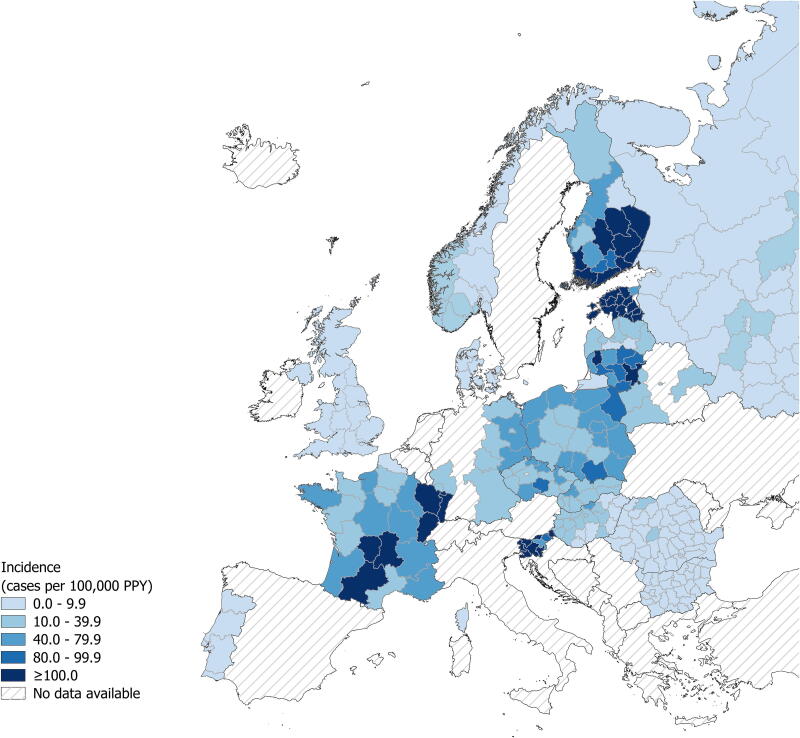
Incidence of surveillance-reported Lyme borreliosis at available subnational levels in Europe during the most recent three-year period. Incidence of LB (cases per 100,000 PPY) during the most recent three-year period from national surveillance systems at the subnational level (where data available). Subnational level displayed varies by country and the most granular regions for which both case and population data could be acquired is shown. Presented national LB surveillance data is of the most recent three years of data available for European countries identified from search methodologies and includes all reporting case classifications as described in Table 1 and Fig. 1. Most recent three years of data vary by subnational region and country. Incidence map of subnational areas of Russia can be found in Supplementary Figure S1. LB, Lyme borreliosis; PPY, population per year.

### Population of Europe living in regions with high LB incidence

Nineteen countries that reported subnational case data were included in the analysis to estimate the minimum percentage of the population in Europe that lives in a region with high LB incidence. Fifty-four percent (183/338) of included subnational regions were considered high incidence for LB (Supplementary Table S3). All subnational regions of Czechia, Estonia, France, Latvia, Lithuania, Poland, Serbia, Slovakia, and Slovenia met the criteria for high LB incidence, indicating that 100% of the populations of these countries live in a high-incidence region. None of the subnational regions of England or Portugal met the criteria, indicating that 0% of the population of those countries lives in a high-incidence region (Fig. 5).

**FIG. 5.**
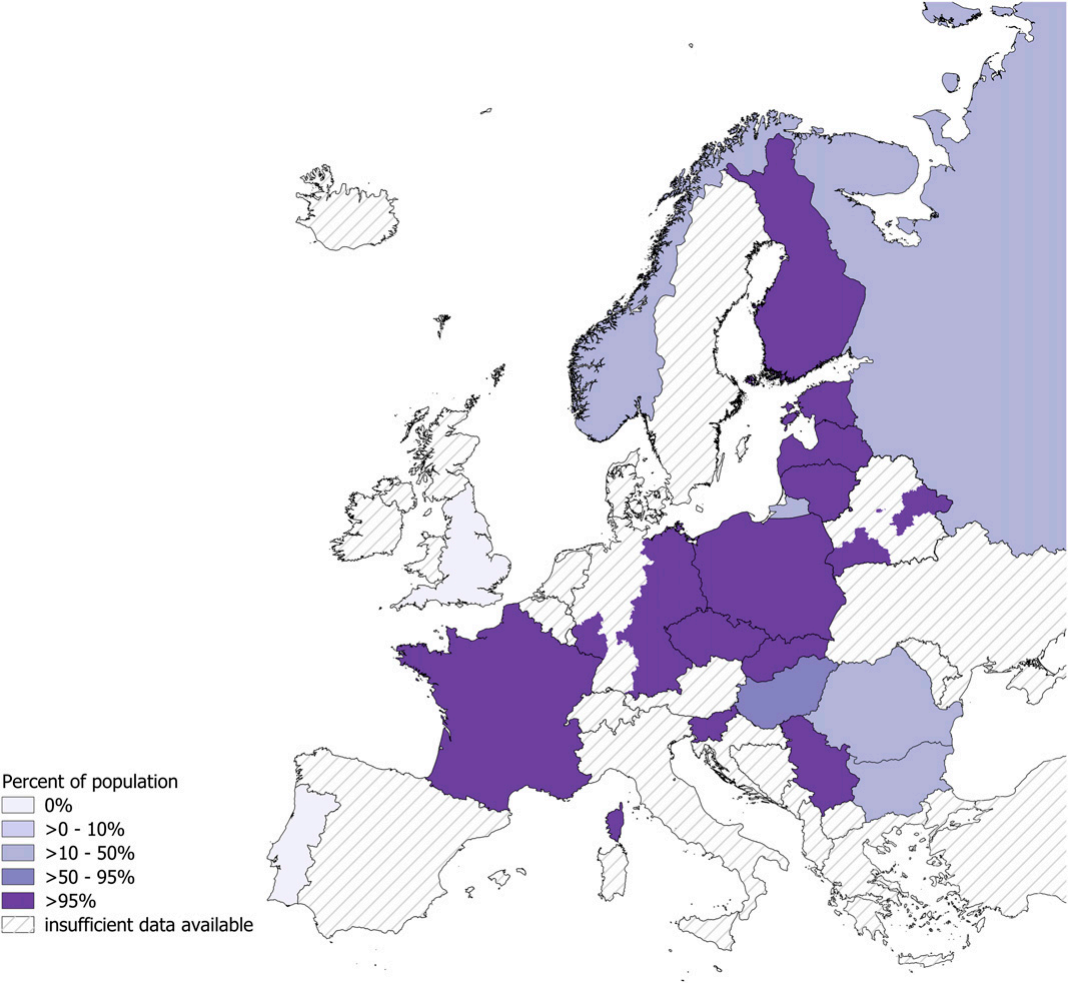
Percent of national European populations living in areas with high Lyme borreliosis incidence. Only countries with subnational data are included. Countries with surveillance data on LNB only are excluded. Subnational regions with high LB incidence are defined as subnational regions that have reported ≥10 cases per 100,000 PPY for any three consecutive year period. Once a region reaches the high-incidence threshold, it remains high-incidence. Percent of population living in a high-incidence subnational region is calculated as the sum of 2023 subnational populations meeting the high-incidence threshold divided by the sum of the country’s subnational populations for which there is LB surveillance data. For Germany and Belarus, there is not surveillance data across all subnational regions, so map includes an estimate only for subnational regions for which there is data. Included regions and calculations are provided in Supplementary Table S3. LNB, Lyme neuroborreliosis; LB, Lyme borreliosis; PPY, population per year.

The 2023 population sum of the 183 high-incidence subnational regions was 222,917,282 and the population sum of all 338 subnational regions of the 19 countries included in this analysis was 432,924,781, resulting in an estimate of at least 51% of the population of these countries living in regions with high LB incidence. Using the same population sum of subnational regions with surveillance data that meet the high-incidence threshold and considering the total 2023 European population of 745,257,338 (https://population.un.org/), a minimum of 30% of all of Europe lives in a region with high LB incidence (Supplementary Table S3).

## Discussion

This review of national surveillance-reported LB cases across Europe is the most comprehensive and updated summary of publicly available case data. Using data across 29 countries, it estimates that an average of nearly 132,000 LB cases, with as many as 173,000 cases, were reported to surveillance systems across Europe each year from 2015 to 2023. Additionally, using surveillance-reported subnational data, this review estimates that a minimum of 30% of the entire population of Europe lives in a high-incidence area for LB.

### Case classification categories for interpreting surveillance data

While differences in case definitions, surveillance system types, and case reporting requirements can make it difficult to compare LB between countries, this updated review classifies countries into five categories based on the types of cases that are included in their publicly available surveillance data, making comparisons of incidence across countries more feasible. Among countries with broader case classifications (EM and laboratory-confirmed LB and EM only), those that make up the most central part of the continent overwhelmingly exhibit higher incidence rates compared with more peripheral countries. Specifically, Finland and Estonia (Northern), Switzerland (Western), and Slovenia (Eastern) reported the highest incidence ≥100 per 100,000 PPY over the last three years. Countries such as Denmark and Norway report lower incidence, but their estimates include only laboratory-confirmed LNB and disseminated LB cases, respectively. However, even with these more restrictive case classifications, their incidence is the same as or higher than several countries with broader case classifications in the U.K. and Ireland and Southern and Eastern Europe. This supports that like other Nordic countries with broader case classifications, Denmark and Norway likely have a high incidence of LB, despite a low rate of reported cases. When comparing surveillance data across countries, it is critical to understand the differences in surveillance methodology and case definitions used by each country to interpret the trends and distribution of LB across Europe.

In addition to using surveillance data to understand geographic disease distribution, it can also be supplemented by other research to better estimate the true LB burden. For example, data on LB seroprevalence and antibody duration have been used to estimate the incidence of symptomatic disease above what is reported by routine surveillance. These studies showed that LB incidence is 2.4–10.5 times higher than what is reported in surveillance in six countries with surveillance systems that capture EM and laboratory-confirmed LB cases and 54.6 and 722.2 times higher in Denmark and Ireland, respectively, where surveillance captures laboratory-confirmed LNB cases only (Angulo et al., 2024). Similar multipliers can be applied to surveillance estimates using data on medically attended LB from sources such as health care claims data, to quantify the number of people seeking medical care for LB. These data can further be used to determine incidence and distributions of LB manifestations, which are rarely reported in surveillance but important in understanding the economic burden of LB (Brestrich et al., 2024a; Geebelen et al., 2022; van den Wijngaard et al., 2017b).

### Influences on recent temporal surveillance trends

On average, LB incidence increased 36% in 18 countries over the most recent two years of reporting. Overall, increases in LB incidence are expected to continue based on increasing risk across Europe. For example, tick populations are expanding to new areas and altitudes due to climate change and warming temperatures (Semenza and Suk, 2018; Voyiatzaki et al., 2022). With this, *Bb* can infect newly vulnerable populations causing LB case numbers to rise rapidly. Persistent monitoring of national and subnational surveillance data over the next several years is necessary not only to track continued increases in LB incidence, but also to identify changes in geographic spread.

Surveillance is a product of its physical environment and the resources that go into it. Understanding the external factors that may impact surveillance is equally important as understanding the data itself. For example, while LB incidence is expected to increase, such increases may not always be observed year to year. In the United States, biannual fluctuations show lower LB incidence in even years than odd years, influenced by the two-year lifecycle of the vector tick (Wormser et al., 2024). Varying biannual fluctuations were also identified in European LB incidence from 2015 to 2023, suggesting this theory may also contribute to observed trends. Other ecological factors such as local weather events and density of tick hosts can also affect observed LB cases from year to year (Giesen et al., 2024).

Additionally, surveillance systems can be temporarily impacted in nonecological and/or unanticipated ways. For example, rates of many reportable diseases decreased during the height of the COVID-19 pandemic, at least partially due to changing health care-seeking behaviors, which led to fewer health care visits (Kuhlen et al., 2020; Tu et al., 2022). Decreases in LB incidence were observed between 2019 and 2021 among 19 of 25 countries that reported LB cases during that time. For example, incidence in Poland and France fell 40% from 2019 to 2021 and 25% from 2020 to 2021, respectively, and these decreases can be partially attributed to direct or indirect effects of COVID-19 (Fig. 2d, e) (Piotrowski and Rymaszewska, 2022; Nuttens et al., 2024). In fact, there is evidence that during the height of the pandemic, social and behavioral changes such as spending more time outside to avoid exposure to COVID-19 likely increased human contact with *Bb-*infected ticks (Borșan et al., 2021; McCormick et al., 2021). Therefore, it is possible that the true number of LB cases increased during this time, but surveillance-reported cases decreased artificially due to the impact of COVID-19 on government resources, health care systems, and care-seeking behaviors. Observed increases in surveillance-reported LB in 2022 and 2023 suggest that surveillance systems have begun to normalize and the increasing trends that were identified prior to COVID-19 are likely to resume (Burn et al., 2023; van den Wijngaard et al., 2017a).

### Strengths and limitations

Differences in LB case definitions and surveillance methods in countries limit direct comparisons of incidence estimates. While documented changes in surveillance reporting were investigated for this review, it is possible that there were changes in surveillance from 2015 to 2023 that were not identified or explicitly described, which could affect interpretation of data. Still, by aggregating country LB data by known case reporting similarities, this review clarifies the groups of countries within which appropriate comparisons can be made. Europe-wide adoption of and adherence to uniform LB reporting practices, such as what ECDC initiated for LNB but considering an even broader scope of disease as proposed by EUCALB, would further improve comparability (European Union, 2018; Stanek et al., 2011). While this requires country buy-in, resources, and coordination, it is advantageous as the disease expands.

Additionally, this review’s key findings of the number of annual cases and the percentage of population living in a high-incidence region in Europe are conservative estimates. Not only do these estimates use LB case numbers that are subject to under-ascertainment but they also do not consider countries without publicly available surveillance data, many of which are known to have high LB incidence, such as Austria, the Netherlands, and Sweden (Stiasny et al., 2021; Hofhuis et al., 2015; Bennet et al., 2006; Dahl et al., 2019). They also do not include countries without surveillance that may have overall low LB rates but may have some regions with high incidence. Furthermore, the estimate for the percentage of population living in high-incidence areas only includes the 66% of countries with national LB surveillance data that provide subnational estimates. Some countries with high-incidence estimates, such as Belgium and Switzerland, are not included in this estimate. Collectively, there is likely much more LB occurring than this review captures.

## Conclusions

Surveillance for LB is conducted and data are made publicly available in 29 European countries. While these surveillance systems have differing case definitions and reporting requirements, this review allows for comparing LB incidence across countries with similar case classifications and highlights higher disease rates in the most central parts of the continent. Overall, this review estimates that 132,000 LB cases are reported to European surveillance systems annually and a minimum of 223 million people, or 30% of the European population, live in a region with high LB incidence. For nearly 70% of countries with recently published LB surveillance, incidence increased on average by 36% across the last two years of reporting. These findings emphasize the need for continued monitoring of LB cases across Europe and new public health prevention tools such as enhanced tick prevention campaigns, improved education among health care providers, and effective vaccines, especially as LB risk is expected to continue increasing in the years to come.

## References

[B1] Angulo FJ, Colby E, Lebech AM, et al. Incidence of symptomatic Lyme borreliosis in nine European countries. Int J Infect Dis 2024;149:107242. 39306075 10.1016/j.ijid.2024.107242

[B2] Bennet L, Halling A, Berglund J. Increased incidence of Lyme borreliosis in southern Sweden following mild winters and during warm, humid summers. Eur J Clin Microbiol Infect Dis 2006;25(7):426–432. 16810531 10.1007/s10096-006-0167-2

[B3] Blanchard L, Jones-Diette J, Lorenc T, et al. Comparison of national surveillance systems for Lyme disease in humans in Europe and North America: A policy review. BMC Public Health 2022;22(1):1307. 35799156 10.1186/s12889-022-13669-w PMC9264653

[B4] Bobe JR, Jutras BL, Horn EJ, et al. Recent progress in lyme disease and remaining challenges. Front Med (Lausanne) 2021;8:666554. 34485323 10.3389/fmed.2021.666554 PMC8416313

[B5] Borșan S-D, Trif SR, Mihalca AD. Recreational behaviour, risk perceptions, and protective practices against ticks: A cross-sectional comparative study before and during the lockdown enforced by the COVID-19 pandemic in Romania. Parasit Vectors 2021;14(1):423. 34425905 10.1186/s13071-021-04944-7 PMC8381130

[B6] Brestrich G, Diesing J, Kossack N, et al. Excess healthcare costs and resource utilisation of lyme borreliosis in Germany: A Propensity Score-Matched Cohort Study. Zoonoses Public Health 2024a;72(1):23–31. 39238143 10.1111/zph.13180 PMC11695699

[B7] Brestrich G, Hagemann C, Diesing J, et al. Incidence of Lyme Borreliosis in Germany: A retrospective observational healthcare claims study. Ticks Tick Borne Dis 2024b;15(3):102326. 38417196 10.1016/j.ttbdis.2024.102326

[B8] Burn L, Tran TMP, Pilz A, et al. Incidence of lyme borreliosis in Europe from national surveillance systems (2005-2020). Vector Borne Zoonotic Dis, 2023;23(4):156–171. 37071405 10.1089/vbz.2022.0071 PMC10122223

[B9] Centers for Disease Control and Prevention. *Lyme Disease (Borrelia burgdorferi) 2022 Case Definition* [Online]. 2021. Available from: https://ndc.services.cdc.gov/case-definitions/lyme-disease-2022/ [Last accessed: November 1, 2024].

[B10] Dahl V, Wisell KT, Giske CG, et al. Lyme neuroborreliosis epidemiology in Sweden 2010 to 2014: Clinical microbiology laboratories are a better data source than the hospital discharge diagnosis register. Euro Surveill 2019;24(20):1800453. 31115310 10.2807/1560-7917.ES.2019.24.20.1800453 PMC6530252

[B11] European Centre for Disease Prevention and Control. A Systematic Literature Review on the Diagnostic Accuracy of Serological Tests for Lyme Borrelios. ECDC: Stockholm; 2016.

[B12] European Centre for Disease Prevention and Control. *Surveillance Atlas of Infectious Diseases* [Online]. European Centre for Disease Prevention and Control; 2024. Available from: https://atlas.ecdc.europa.eu/public/index.aspx [Last accessed: November 25, 2024].

[B13] European Union. Commission implementing decision (EU) 2018/945 of 22 June 2018 on the communicable diseases and related special health issues to be covered by epidemiological surveillance as well as relevant case definitions. Official Journal of the European Union, 2018. 170.

[B14] Geebelen L, Devleesschauwer B, Lernout T, et al. Lyme borreliosis in Belgium: A cost-of-illness analysis. BMC Public Health 2022;22(1):2194. 36443755 10.1186/s12889-022-14380-6 PMC9703731

[B15] Geebelen L, VAN Cauteren D, Devleesschauwer B, et al. Combining primary care surveillance and a meta-analysis to estimate the incidence of the clinical manifestations of Lyme borreliosis in Belgium, 2015–2017. Ticks Tick Borne Dis 2019;10(3):598–605. 30772196 10.1016/j.ttbdis.2018.12.007

[B16] Giesen C, Cifo D, Gomez-Barroso D, et al. The role of environmental factors in lyme disease transmission in the European Union: A systematic review. Trop Med Infect Dis 2024;9(5):113. 38787046 10.3390/tropicalmed9050113 PMC11125681

[B17] Hofhuis A, Harms M, Bennema S, et al. Physician reported incidence of early and late Lyme borreliosis. Parasit Vectors 2015;8:161. 25889086 10.1186/s13071-015-0777-6 PMC4363353

[B18] Janova E. Emerging and threatening vector-borne zoonoses in the world and in Europe: A brief update. Pathog Glob Health 2019;113:49–57. 30916639 10.1080/20477724.2019.1598127 PMC6493274

[B19] Kuhlen R, Schmithausen D, Winklmair C, et al. The effects of the COVID-19 pandemic and lockdown on routine hospital care for other illnesses. Dtsch Arztebl Int 2020;117(27–28):488–489. 33050998 10.3238/arztebl.2020.0488 PMC7575899

[B20] Mccormick DW, Kugeler KJ, Marx GE, et al. Effects of COVID-19 pandemic on reported lyme disease, United States, 2020. Emerg Infect Dis, 2021;27(10):2715–2717. 34545801 10.3201/eid2710.210903 PMC8462321

[B21] Nagarajan A, Skufca J, Vyse A, et al. The landscape of lyme borreliosis surveillance in Europe. Vector Borne Zoonotic Dis, 2023;23(4):142–155. 37071402 10.1089/vbz.2022.0067 PMC10122255

[B22] Nuttens C, Duret S, Bessou A, et al. Evolution of lyme borreliosis epidemiology in primary care and hospital settings in france during the COVID-19 pandemic. Vector Borne Zoonotic Dis 2024;24(11):720–729. 39013022 10.1089/vbz.2024.0006

[B23] Piotrowski M, Rymaszewska A. The impact of a pandemic COVID-19 on the incidence of borreliosis in Poland. Acta Parasitol 2022;67(2):1007–1009. 34978679 10.1007/s11686-021-00495-0 PMC8721174

[B24] Semenza JC, Suk JE. Vector-borne diseases and climate change: A European perspective. FEMS Microbiol Lett 2018;365(2):fnx244. 29149298 10.1093/femsle/fnx244 PMC5812531

[B25] Stanek G, Fingerle V, Hunfeld KP, et al. Lyme borreliosis: Clinical case definitions for diagnosis and management in Europe. Clin Microbiol Infect 2011;17(1):69–79. 20132258 10.1111/j.1469-0691.2010.03175.x

[B26] Stanek G, Strle F. Lyme borreliosis-from tick bite to diagnosis and treatment. FEMS Microbiol Rev 2018;42(3):233–258. 29893904 10.1093/femsre/fux047

[B27] Steere AC. Lyme disease. N Engl J Med 2001;345(2):115–125. 11450660 10.1056/NEJM200107123450207

[B28] Steere AC, Strle F, Wormser GP, et al. Lyme borreliosis. Nat Rev Dis Primers 2016;2:16090. 27976670 10.1038/nrdp.2016.90 PMC5539539

[B29] Stiasny K, Santonja I, Holzmann H, et al. The regional decline and rise of tick-borne encephalitis incidence do not correlate with Lyme borreliosis, Austria, 2005 to 2018. Euro Surveill 2021;26(35):2002108. 34477056 10.2807/1560-7917.ES.2021.26.35.2002108 PMC8414957

[B30] Tu K, Sarkadi Kristiansson R, Gronsbell J, et al.; INTREPID. Changes in primary care visits arising from the COVID-19 pandemic: An international comparative study by the International Consortium of Primary Care Big Data Researchers (INTRePID). BMJ Open 2022;12(5):e059130. 10.1136/bmjopen-2021-059130 PMC9086267 35534063

[B31] VAN DEN Wijngaard CC, Hofhuis A, Simoes M, et al. Surveillance perspective on Lyme borreliosis across the European Union and European Economic Area. Euro Surveill 2017a;22(27):30569. 28703098 10.2807/1560-7917.ES.2017.22.27.30569 PMC5508331

[B32] VAN DEN Wijngaard CC, Hofhuis A, Wong A, et al. The cost of Lyme borreliosis. Eur J Public Health 2017b;27(3):538–547. 28444236 10.1093/eurpub/ckw269

[B33] Voyiatzaki C, Papailia SI, Venetikou MS, et al. Climate changes exacerbate the spread of Ixodes ricinus and the occurrence of lyme borreliosis and tick-borne encephalitis in europe-how climate models are used as a risk assessment approach for tick-borne diseases. Int J Environ Res Public Health 2022;19(11):6516. 35682098 10.3390/ijerph19116516 PMC9180659

[B34] World Health Organization. *Annex: Regional Classifications* [Online]. 2024. Available from: https://www.who.int/observatories/global-observatory-on-health-research-and-development/classifications-and-standards/country-groupings [Last accessed: November 1, 2024].

[B35] Wormser GP. Clinical practice. Early lyme disease. N Engl J Med 2006;354(26):2794–2801. 16807416 10.1056/NEJMcp061181

[B36] Wormser GP, Schneider LM, Joseph JT, Fish D. Surveillance data from 2011-2020 indicate a lower risk of Lyme disease in the USA in even-numbered years. Wien Klin Wochenschr 2024;136(21–22):636–638. 39352479 10.1007/s00508-024-02452-z

