# Supplementary material for: Lyme Borreliosis Incidence Across Europe, 2015–2023: A Surveillance-Based Review and Analysis
Source: Vector Borne Zoonotic Dis. 2025 Oct 3;25(10):569–79. doi: 10.1177/15303667251363125 (PMC12629682; doi:10.1177/15303667251363125)
Supplement: Supplementary file 1 [file 15303667251363125_suppl_figures.docx]

**Figure S1. Incidence of surveillance-reported Lyme borreliosis at subnational levels of Russia during the most recent three-year period, 2021-2023**

**Fig S1.** Incidence of LB (cases per 100,000 PPY) during 2021-2023 at Russia’s subnational regions as reported in Russia’s national surveillance reports. Presented subnational LB surveillance data is of the most recent three years of data available for Russia and includes both EM and laboratory-confirmed LB cases. EM = erythema migrans, LB = Lyme borreliosis, PPY = population per year.

**Figure S2. Incidence of surveillance-reported Lyme borreliosis by country at the most granular geographic area available (subnational or national) in Europe during the most recent three-year period**

**Fig S2.** Incidence of LB (cases per 100,000 PPY) during the most recent three-year period from national surveillance systems at the most granular geography area, national or subnational level, identified in public surveillance reports. Presented LB surveillance incidence estimates are of the most recent three years of data available for each subnational region and country identified from search methodologies. Most recent three years of data varies by subnational region and country. Incidence map of subnational areas of Russia can be found in Figure S1. LB = Lyme borreliosis, PPY = population per year.
